# Subarachnoidal Neurocysticercosis non-responsive to cysticidal drugs: a case series

**DOI:** 10.1186/1471-2377-10-16

**Published:** 2010-03-04

**Authors:** Graciela Cárdenas, Roger Carrillo-Mezo, Helgi Jung, Edda Sciutto, Jose Luis Soto Hernandez, Agnès Fleury

**Affiliations:** 1 Instituto Nacional de Neurología y Neurocirugía, Insurgentes Sur 3877, Colonia La Fama, Delegación Tlalpan, México DF, México, CP 14269; 2 Instituto de Investigaciones Biomédicas, Universidad Nacional Autónoma de México, Departamento de Inmunología, AP70228, México DF 04510, México

## Abstract

**Background:**

Neurocysticercosis (NC) is one of the most frequent parasitic diseases of the central nervous system. Cysticidal drugs, albendazole and praziquantel, are generally effective when parasites localize in the parenchyma. In contrast, parasites lodged in the subarachnoid basal cisterns are less responsive to treatment.

**Case Presentation:**

The clinical and radiological pictures of six Mexican patients non-respondent to cysticidal treatment are presented.

**Conclusions:**

The possible factors involved in the cysticidal non-response are discussed and hints are provided of potentially useful changes to therapeutic protocols.

## Background

*Taenia solium *is a parasite which larvae (cysticercus) may localize in the central nervous system of humans causing neurocysticercosis (NC). Most NC cases occur with little or no neurological symptoms but others may present a variety of non-specific mild clinical symptoms (headache, partial seizures) or severe neurological syndromes with intracraneal hypertension and generalized seizures [1].

The introduction of cysticidal drugs, albendazole (ABZ) and praziquantel (PZQ), for the treatment of NC have dramatically improved its prognosis [2]. In Mexico, ABZ is becoming the drug of choice due to its low costs and availability. Parasite localization is one of the main factors involved in the success of the treatment. When cysticerci are lodged in the parenchyma, the use of these drugs allows generally a prompt radiological and clinical improvement in most of the patients although only a modest effect is reported in some cases [3,4]. When the parasites are located in the subarachnoid basal cisterns (SA-NC), the prognosis is more uncertain. Several case series have reported the effectiveness of these drugs in SA-NC treatment [5-7]. However, in our experience and that of others [4], it is a frequent finding that the parasites persist after treatment, even when a high dose of ABZ has been used [8].

In this report, six cases NC-SA patients who are non-respondent to conventional pharmacological treatment and a brief review of literature are described.

## Case Presentation

**Figure 1:**
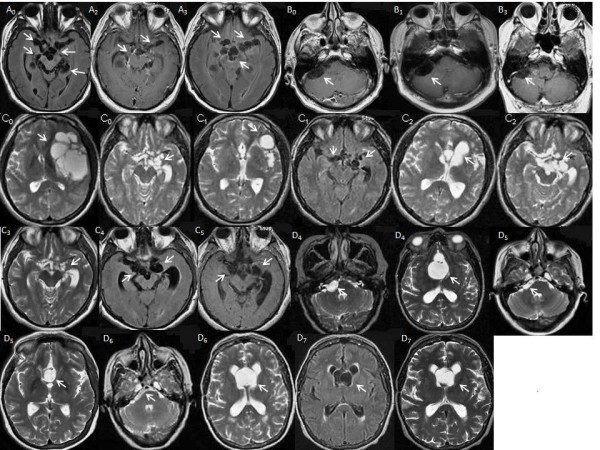
**MRI of patients**. Letters correspond to each of the patients and numbers corresponds to the moment of the MRI in relation to the number of cysticidal cycles administered (0: basal MRI, 1: after the first cycle, etc.). A0 (MRI T2 FLAIR): vesicular cysticerci in basal cisterns. Decrease (A2, MRI CE, T1) and increase (A3, MRI T2 FLAIR) in parasites. B0 (MRI CE T1): Vesicular parasites in the pontocerebellar angle. B1: Increase of parasite. B3: Persistence of parasite. C0 (MRI T2): Cysticercus in the Sylvian and basal cisterns. C1 (MRI T2, FLAIR): Decrease in Sylvian and persistence of basal cisterns parasites. C2 (MRI T2): Increase of lamina terminalis and basal cisterns parasites. C3 (MRI T2): Decrease of basal cisterns parasites. C4 (MRI CE T1): Increase of basal cisterns parasites and appearance of new ones. C5 (MRI FLAIR): Persistence of parasites. D4 (MRI T2): Parasites in the right pontocerebellar angle and in the lamina terminalis cisterns. D5 (MRI T2): Decrease of parasite volume. D6 (MRI T2): Increase of the lamina terminal cistern vesicles. D7 (MRI FLAIR, T2): Persistence of vesicles.

Patient A (Figure 1, A0 to 1A3). A 46-year-old man with one year history of increasing incapacitating frontal headache. Upon admission, bilateral papilledema and upward gaze paralysis were shown. Multiple vesicular cysticerci located in opto-chiasmatic and perimensencephalic cisterns as well as basal meningeal enhancement were observed by magnetic resonance imaging (MRI). Increased cellularity (89 cells/mm3) and anti-cysticercal antibodies (Abs) determined by ELISA were detected in the cerebrospinal fluid (CSF). During one year, the patient received two cycles of ABZ (30 mg/kg/day) and one course of PZQ (50 mg/kg), for eight days, associated to corticosteroids. In the ninth month, he required a ventriculoperitoneal shunt (VPS) due to hydrocephaly. In all of MRIs, which were performed four months after each treatment cycle, most of the vesicular parasites persisted.

Patient B (Figure 1, B0 to 1B3). A 60 year old man with chronic headache. Nine months before hospital admission, headache frequency increased and lower limb motor dysfunction appeared. Neurological examination revealed only papilledema. On MRI, vesicular subarachnoidal NC in posterior fossa was observed. CSF analysis showed an inflammatory profile with abundant lymphocytes and eosinophils. During the year following the diagnosis, he received two courses of ABZ at 30 mg/kg/day during 8 days and one course of ABZ at the same dose, associated with PZQ at 50 mg/kg plus corticosteroids. However, radiological evidence of vesicular parasites was still observed and CSF inflammatory profile and clinical complaints persisted.

Patient C (Figure 1, C0 to 1C5). A 29-year old man with new onset generalized seizures. CT scan showed vesicular NC and he received ABZ plus steroids in the United States of America. Post treatment, progressive motor dysphasia, right sided weakness and intracranial hypertension signs appeared. The patient returned to Mexico. MRI showed vesicular racemose cysticercosis on left Sylvian fissure with displacement of midline anatomical structures. A second ABZ cycle was administered and although the racemose cyst evinced a substantial reduction, other SA vesicular parasites appeared. During two years this patient received four other courses of ABZ (30 mg/kg/day during eight days) and two combined courses of ABZ and PZQ. These treatments were delivered at the same doses as with the other patients and showed no evidence of parasite eradication.

Patient D (Figure 1, D4 to 1D7). A 65-year-old woman with vomiting, papilledema, gait disturbances, and urinary incontinency. The patient had a 6-year history of chronic headache. On admission, CT scan showed multiple vesicular parasites in the Sylvian fissure with mass effect. A surgical removal of parasites located in the right Sylvian fissure and opto-chiasmatic cistern was performed. Three months after surgical procedure, a CT scan showed persistence of subarachnoid vesicular parasites in the chiasma and pontocerebellar cistern. CSF analysis revealed an inflammatory profile (20 cells/mm^3^) with presence of anti-cysticercal Abs. During four years, patient completed seven courses of ABZ (30 mg/kg/day during 8 days) associated to corticosteroids without complete clinical and radiological improvement.

**Figure 2:**
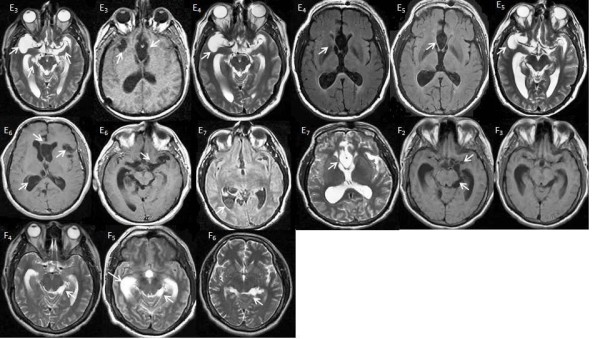
**MRI of patients**. E3 (MRI T2, T1): Parasites in Sylvian, basal and lamina terminalis cisterns. E4 and E5 (MRI T2, FLAIR): Persistence of some of the parasites. E6 (MRI simple, CE T1): Persistence of lamina terminalis parasites and appearance of new ones. E7 (MRI FLAIR, T2): Persistence of parasites. F2 (MRI T1): Parasites in the crural and ambient cisterns. F3 (MRI T1), F4, F5 and F6 (MRI T2): Persistence of ambient cistern parasites.

Patient E (Figure 2, E3 to 2E7). A 40-year old man with a one-year-history of increasing headache, in frequency and intensity, associated with sporadic vomiting and generalized seizures. Papilledema and left slight motor weakness were observed on neurological examinations. A CT scan revealed a vesicular racemose cysticercus on the right Sylvian fissure, upon which surgical excision of parasites was performed. Diagnosis of vesicular cysticerci was confirmed by histopathology. Post-treatment, the patient showed clinical improvement, but six months later, motor deficit reappeared. MRI showed recurrence of parasites at the same location and CSF was inflammatory. During the five years following the first surgery, the parasites did not disappear in spite of the administration of seven courses of ABZ and one combined course of ABZ and PZQ with corticosteroids. Clinically, the patient presents memory loss and corticosteroids side-effects as hyperglycemia and myopathy.

Patient F (Figure 2, F2 to 2F6). A 26-year old man with new onset partial motor epilepsy. His CT scan showed perimesencephalic vesicular parasites and CSF was inflammatory (75 cells/mm^3^) with specific anticysticercal Abs. During the following five years, the patient received 7 courses of ABZ (30 mg/kg/day during eight days with corticosteroids) and he required the placement of a VPS for hydrocephalus during the fourth year. In spite of the received treatment, vesicular parasites persisted on the last MRI, and CSF was still inflammatory.

## Conclusion

This article reports six SA-NC patients in whom parasites relapsed despite repetitive administration of cysticidal treatment. Other non-responsive NC cases have been previously published (Table 1, [9-12]) but including patients that differ in the clinical picture, the parasite localization and cysticidal doses used. Even though the causes of the parasite's persistence, despite the ABZ treatment, are still unknown, multiple host and parasite factors may be involved. The possibility that the cysticidal drugs differentially affect the parasites according to their developmental stage (radiologically not- definable) could be a factor involved in the non-response observed. It is also possible that the lesser penetration of ABZ in the subarachnoidal space could participate in the SA-NC resistance. Another factor involved in this phenomenon could be related to the variability in plasma and CSF ABZ sulphoxide levels among patients due to individual differences in bioavailability [2]. The high levels of corticosteroids used to prevent complications due to severe CSF inflammation may also turn-off key immunological components crucial for the parasite destruction. Finally, although resistance of cysticerci to ABZ in humans has never been reported, it may occur as it has been observed in other related parasites [13-15].

**Table 1:** Previous published cases of non-responder patients to cysticidal drugs.

Patient	Age/Sex	Parasites location	Clinical manifestations	Treatment	Reference
1	26/M†	SA (sulci)	Generalized seizures	PZQ, ABZ	[9]
2	38/F	SA (sulci)	Generalized seizures	PZQ, PZQ + ABZ	[10]
3	44/M	SA (Sylvian fissure)	Generalized seizures	ABZ, ivermectine	[11]
4	69/M	SA (sulci)	Partial seizures	ABZ, ivermectine	[11]
5	45/F	Fourth ventricle	Intracranial hypertension and ataxia	ABZ, ABZ + ivermectine	[11]
6	37/M	SA (sulci)	Partial seizures	ABZ, PZQ + ABZ, ivermectine	[11]
7	38/F	SAb and SA sulci	Seizures, hydrocephalus and motor deficit	ABZ	[12]

†M: male, F: female

Whatever are the reasons, this scenario clearly points out to the relevance of developing new therapeutic strategies. One approach could be the elaboration of new albendazole formulations in order to improve its bioavailability and therapeutic efficacy [16]. Another approach is the search of new pharmacological alternatives as ivermectine, which seems to destroy cysticerci in patients resistant to cysticercidal drugs [11]. Nitazoxanide and tizoxanide combined with ABZ have also shown promising results albeit in vitro and using another related cestode [17].

Finally, although these non-respondent cases are not the most frequent, their severity should encourage controlled studies to evaluate new forms of medical intervention and management. In particular, fuller understanding of the key elements that participate in controlling the inflammatory response would greatly help in devising new, potent and less harmful ways to prevent severe disease.

## Consent

Written informed consent was obtained from the patients for publication of these cases report and any accompanying images. A copy of the written consents is available for review by the Editor-in-Chief of this journal.

## Competing interests

The authors declare that they have no competing interests.

## Authors' contributions

GC has been involved in the acquisition of clinical data and in drafting the manuscript. RCM has been involved in the acquisition of radiological data and in drafting the radiological part of the manuscript. HJ has been involved in the revision of the manuscript critically for important intellectual content. ES has been involved in the revision of the manuscript critically for important intellectual content. JLSH has been involved in the acquisition of clinical data, and in revision of manuscript for important intellectual content. AF participated in the design and coordination of the study, in drafting the manuscript and in its revision for important intellectual content. All authors read and approved the final manuscript.

## Pre-publication history

The pre-publication history for this paper can be accessed here:

http://www.biomedcentral.com/1471-2377/10/16/prepub
